# The use of systemic immune-inflammation index in the differential diagnosis of acute scrotum in children

**DOI:** 10.12669/pjms.42.4.14849

**Published:** 2026-04

**Authors:** Hasan Madenci, Cuneyt Ugur

**Affiliations:** 1 Hasan Madenci, MD. Department of Pediatric Surgery, Faculty of Medicine, University Necmettin Erbakan, Konya, Turkiye; 2 Cuneyt Ugur, MD. Associate Professor, Department of Pediatrics, University of Health Sciences Turkey, Konya City Health Application and Research Center, Konya, Turkiye

**Keywords:** Acute scrotum, C-reactive protein, Epididymo-orchitis, Scrotal doppler ultrasonography, Systemic immune-inflammation index, Testicular torsion

## Abstract

**Background & Objectives:**

Acute scrotum is an emergency condition characterized by scrotal pain, swelling, and erythema. While testicular torsion requires emergency surgery, epididymo-orchitis requires medical treatment. In our study, we investigated the utility of the systemic immune-inflammation index in the differential diagnosis of acute scrotum in children. Our objective was to see if we can we use SII in the differential diagnosis of epididymal orchitis and testicular torsion by comparing retrospective data of patients diagnosed with acute scrotum who applied to our pediatric emergency clinic?

**Methodology:**

In our study, we retrospectively reviewed the medical records of patients who presented to our emergency department (Konya City Hospital / Turkiye) with complaints such as testicular pain, swelling, and redness between December 2012 to December 2022.Testicular torsion (TT) and epididymo-orchitis (EO) groups were formed among the patients admitted to our pediatric emergency outpatient clinic with complaints of the acute scrotum and treated. Scrotal doppler ultrasonography (DUSG), C-reactive protein (CRP), white blood cell (WBC), neutrophil (NE), lymphocyte (LY), monocyte (MO), and platelet (PLT) values of patients were recorded.

**Results:**

No statistically significant difference was found between TT and EO groups in terms of age, DUSG, WBC, NEU, LY, MO, and PLT. There was a significant difference between the groups in terms of CRP. CRP was found to be significantly higher in the EO group compared to the TT group (P<0.001). TT group was analyzed within itself and scrotal DUSG was found to be valuable in diagnosis. There was no significant difference between the groups in terms of SII value.

**Conclusion:**

Parameters such as CRP and scrotal DUSG, which are commonly used in the differential diagnosis of AS, are still valuable. More studies are needed to determine the use of SII in the differential diagnosis of AS.

## INTRODUCTION

Acute scrotum (AS) is an emergency condition characterized by scrotal pain, swelling, and erythema.^1^ The most common causes in childhood are testicular torsion (TT) (20-30%), appendiceal testicular torsion (40-74%), epididymo-orchitis (EO) (5-15%), idiopathic scrotal edema and trauma (10%).^1^,^2^ While EO can be treated medically, TT requires urgent surgical intervention. Testicular viability is inversely proportional with time between the onset of torsion and intervention.^3^ Ascending infections are usually blamed for the pathogenesis of epididymoorchitis, and the etiology of testicular torsion is unknown.^3^,^4^ The longer the time until detorsion, the more testicular viability will decrease.^4^,^5^

History, physical examination, and, if necessary, scrotal doppler ultrasonography (DUSG) should be evaluated together for the differential diagnosis of AS.^6^ Due to some reasons such as the lack of DUSG in many centers, the inability to perform DUSG outside certain hours of the day, and the lack of experienced staff and child compliance, tests giving rapid results and examinations with high diagnostic value are still needed in the differential diagnosis.

Systemic immune-inflammation index (SII) was defined as (neutrophil count x platelet count /lymphocyte count) by Hu et al., in 2014.^7^ While SII was initially assessed for the determination of prognosis mostly in cancer, it has been used as the acute indicator of inflammation today.^8^-^10^ EO is generally a bacterial-origin infection, and it is treated with antibiotics. Therefore, it is expected for the acute infection values to increase in blood in EO. In torsion, as the essential pathology is circulatory impairment, the clinical picture is characterized by ischemia and an increase of infection values in the blood is not expected.^3^,^4^ The aim of the present study was to investigate the usability of SII in the differential diagnosis of EO and TT. To the best of our knowledge, there are no studies in the literature on this matter.

## METHODOLOGY

In the present study, the medical records of patients who were admitted to the hospital with AS findings such as testicular pain, swelling, and erythema between December 2012 to December 2022 were retrospectively reviewed.

### Ethical approval:

The study was performed with the approval of the Scientific Research Ethics Committee XX (Dated: October 26, 2022 and numbered 13).

### Inclusion criteria:

Patients, who were younger than 18 years of age and had a definitive diagnosis of EO or TT were included in the study.

### Exclusion criteria:

Cases with appendiceal testicular torsion, those with a preliminary diagnosis of TT who were not operated (patients who were detorsioned in company with US or patients who did not accept the operation), those suffering from any infectious disease in the last month, cancer patients, those with a history of systemic inflammatory disease, and those using steroids or immunosuppressive drugs were excluded from the study.

### Study population:

EO Group: This group consisted of the patients who were diagnosed with EO and whose treatment was completed medically. EO diagnosis was made by clinical, laboratory and DUSG. It was observed that EO cases were treated as in-patients or out-patients. It was determined that out-patients were examined again and evaluated on the 2^nd^ or 3^rd^ day of treatment.

### TT group:

This group consisted of the patients who were operated on with the preliminary diagnosis of TT and whose diagnosis was confirmed during surgery.

Scrotal DUSG, C-reactive protein (CRP), white blood cell (WBC), neutrophil (NE), lymphocyte (LY), monocyte (MO), and platelet (PLT) values were recorded retrospectively. In addition, neutrophil-to-lymphocyte ratio (NLO), monocyte-to-lymphocyte ratio (MLO), platelet-to-lymphocyte ratio (PLO), and SII were calculated. In addition, the time from the onset of the complaint to the operation, the operation method, anesthesia technique, the location of the torsion, and the number of turns of the testicle were recorded in the torsion group. Blood tests were performed on venous blood samples taken from patients at the time of admission to emergency department.

Among 45 patients who were operated on with the preliminary diagnosis of TT, seven patients (appendiceal testicular torsion in five cases and EO in two cases) were not torsion and they were not included in the TT group. These two EO cases were added to the 36 patients in EO group and 38 patients were included in the study in EO group. Thus, the data of 38 patients in both patient groups were recorded. TT group was divided into two subgroups in terms of the time between the onset of complaints and the time of intervention. Testicular Torsion 1 (TT1): Patients who were intervened in the first six hours after the onset of complaints. There were 15 patients in this group. Testicular Torsion 2 (TT2): Patients who were intervened more than six hours after the onset of complaints. There were 23 patients in this group.

### Statistical Analysis:

Descriptive statistics methods were used. Normality Test, including Kolmogorov-Smirnov and Shapiro-Wilk tests, was used to determine the data distribution. Data with a normal distribution were expressed as mean±standard deviation and non-normally distributed data were expressed as median (25^th^- 75^th^ percentile). Categorical variables were expressed as numbers (n) and percentages (%). Student t-test and Mann-Whitney U test were used for the comparison of numerical data between groups. Chi-square test was used to compare categorical variables. Statistical Package for Social Sciences (SPSS) Windows software (ver. 22; IBM SPSS, Chicago, USA) was used for all statistical analyses. P value of < 0.05 was considered as statistically significant.

## RESULTS

There were 76 patients in the present study, 38 were included in the TT group and 38 in the EO group. The median age distribution of the groups was 13.00 (11.00-15.00) years in the EO group and 14.00 (12.75-15.00) years in the TT group. There was no statistically significant difference between the groups in terms of age (P=0.104). The mean WBC counts were 11.37±3.10 10³/ μL in the TT group and 10.92±3.35 10³/ μL in the EO group, and there was no significant difference between the groups in terms of WBC (P=0. 543). The median NE counts of the groups were 7.25 (4.81-9.65) 10³/ μL and 5.98 (4.62-9.59) 10³/ μL and there was no significant difference between the groups in terms of NE (P=.0.424). There was no significant difference between the groups in terms of LY, MO, and PLT counts. The median CRP value was 3.03 (1.07-3.27) mg/L in the TT group and 6.07 (2.23-16.03) mg/L in the EO group. There was a statistically significant difference between the groups in terms of CRP value (P<0.001) .CRP was found to be significantly higher in the EO group compared to the TT group. The SII value was 672.46 (486.50-1049.30) for the TT group and 652.33 (422.10-1187.83) for the EO group. No significant difference was found between both groups in terms of SII value (P=0.795) (Table-I).

**Table-I:** Demographic characteristics and laboratory findings of testicular torsion and epididymo-orchitis groups.

	Testicular Torsion n=38	Epididymo-orchitis n=38	p
Age (Year)	14.00(12.75-15.00)	13.00(11.00-15.00)	0.104^x^
WBC(10³/ μL)	11.37 ±3.10	10.92±3.35	0.543^y^
Neutrophil (10³/ μL)	7.25 (4.81-9.65)	5.98 (4,62-9.59)	0.424^x^
Lymphocyte (10³/ μL)	7.25 (4.81-9.65)	5.9750 (4.64-9.60)	0.540^x^
Monocyte (10³/ μL)	0.805 (0.52-1.02)	0.805 (0.66-1.01)	0.648^x^
Platelet (10³/ μL)	310.68 ± 65.23	318.47± 77.51	0.637^y^
CRP (mg/L)	3.03 (1.07-3.27)	6.07 (2.23-16.03)	<0.001^x^
NLR	2.21 (1.13-4.04)	1.96(1.32-3.65)	0.779^x^
MLR	0.27 (0,16-0,37)	0.25 (0.20-0.45)	0.432^x^
PLR	105.18 (80.26-136.16)	120.48 (86.92-151.42)	0.269^x^
SII	672.46 (486.50-1049.30)	652.33(422.10-1187.83)	0.795^x^
***Side***			
Left	23 (60.5)	20 (52.6)	0.269^z^
Right	15 (39.5)	15(39.5)	
Bilateral	-	3 (7.9)	
***Ultrasonography***			
Positive	34 (89.5)	36 (94.7)	
Negative	4 (0.5)	2 (5.3)	
			0.674^z^

x: Mann-Whitney U y: Student’s T test z: Ki-kare test Note: Parameters are expressed as median (25^th^-75^th^ percentile), mean ± standard deviation and n (%). x: Mann-Whitney U. y: Student’s T test. Explanation: WBC, white blood cell; CRP, c-reactive protein; NLR, neutrophil-to-lymphocyte ratio; PLR, platelet-to-lymphocyte ratio; SII, systemic immune-inflammation index. Note: Parameters are expressed as median (25^th^-75^th^ percentile) and mean ± standard deviation.

While 23 left testicular involvement and 15 right testicular involvement were observed in the TT group, 20 left testicular involvement, 15 right testicular involvement, and three bilateral testes involvement were observed in the EO group. There was no significant difference in terms of side involvement. DUSG results were diagnostic in 34 patients in the TT group and in all 38 patients in the EO group (Table-I).

When TT group is examined in itself, the median time from the onset of complaints to the operation was 8.00 (4.75-24.00) hours. Among operated patients, detorsion-fixation surgery was performed in 15 patients, detorsion in 17, orchiectomy in 5, and orchiopexy-detorsion-fixation in one patient. In 27 of the patients operated on for TT, the scrotum was approached through a midline incision, and 11 patients were operated on with an inguinal transverse approach. While general anesthesia was used in 32 patients, spinal anesthesia was used in six patients. The site of torsion was intravaginal in 33 patients and extra-vaginal in five patients. The testis was rotated two turns in 23 patients, one turn in 13 patients, and ≥3 turns in two patients (Table-II).

**Table-II:** Characteristics of the testicular torsion group regarding the operation.

Time of operation (hrs)	8.00 (4.75-24.00)
***Operation performed***	
Detorsion fixation	15 (39.5)
Detorsion	17 (44.7)
Orchiectomy	5 (13.2)
Orchiopexy-detorsion-fixation	1 (2.6)
***Site of incision***	
Scrotum midline	27 (71.1)
Inguinal transverse	11 (28.9)
***Anesthesia technique***	
General	32 (84.2)
Spinal	6 (15.8)
***Site of torsion***	
Intravaginal	33 (86.8)
Extra-vaginal	5 (13.2)
***No. of turns***	
1	13 (34.2)
2	23 (60.5)
≥3	2 (5.3)

***Note:*** Parameters are given as median (25^th^-75^th^ percentile) and n (%).

No significant difference was found between TT1 and TT2 groups in terms of age, hematological parameters, CRP, and SII. Table-III shows the results obtained by comparing the TT1 and TT2 groups within themselves.

**Table-III:** Demographic characteristics and laboratory findings according to the subgroups of testicular torsion.

	Testicular Torsion 1 n=15	Testicular Torsion 2 n=23	P
Age (Year)	14.00(13.50-14.50)	14.00(12.00-15.50)	.768^x^
WBC(10³/ μL)	12.21 ±2.91	10.82±3.16	.181^y^
Neutrophil (10³/ μL)	8.38± 3.05	6.70± 2.84	.095^y^
Lymphocyte (10³/ μL)	2.86(1.76-3.31)	2.96 (2.54-4.27)	.344^x^
Monocyte (10³/ μL)	0.83 (0.45-1.18)	0.74 (0.58-0.97)	.836^x^
Platelet (10³/ μL)	302.33 ± 65,42	316,13± 65.98	.531^y^
CRP (mg/L)	2.05 (1.15-3.20)	3.11 (1.54-3.25)	.516^x^
NLR	3.41 (1.97-477)	1.91(1.30-2.92)	.153^x^
MLR	0.31 (0,16-0,37)	0.26 (0.18-0.31)	.516^x^
PLR	106.85 (90.98-1149.32)	102.36 (77.13-130.17)	.535^x^
SII	945.26 (598.41-1333.28)	606.77 (446.06-959.85)	.224^x^

x: Mann-Whitney U. y: Student’s T test. Explanation: WBC, white blood cell; CRP, c-reactive protein; NLR, neutrophil-to-lymphocyte ratio; PLR, platelet-to-lymphocyte ratio; SII, systemic immune-inflammation index. Note: Parameters are expressed as median (25^th^-75^th^ percentile) and mean ± standard deviation.

## DISCUSSION

In our hospital, during testicular torsion surgery: if the testicular nutrition of the torsioned testicle is improved during surgery, we perform fixation to the ipsilateral testicle; if the testicular nutrition is not improved during surgery and testicular oriectomy will not be performed, we perform fixation to the ipsilateral testicle and the opposite testicle; if the patient has undergone oriectomy, we perform fixation to the opposite testicle.

Testicular torsion is the most common pediatric urological emergency.^8^,^11^ The effect of the time until surgical intervention on testicular viability in torsion is known.^5^ Circulatory disturbance caused by rotation of the vascular pedicle leads to ischemia-reperfusion (IR) damage and may cause loss of testicular tissue. Experimental animal studies have shown that 720 degrees of torsion damages the testicular seminiferous epithelium.^12^ IR damage leads to a decrease in the number of germ cells, reduced sperm production, and germ cell apoptosis by recruitment of neutrophils out of the venule and increasing reactive oxygen radicals.^13^ In order to prevent this damage, torsion should be rapidly diagnosed and separated from medically treated EO, and surgical exploration is even recommended in suspected cases.^14^

In the recent years, studies were published showing that SII is beneficial in the diagnosis and follow-up of many inflammatory diseases.^15^-^17^ In the current study, no significant statistical difference was found in terms of SII values between both groups, although TT and EO developed through two similar but different inflammatory processes.

The reflection of infective processes of EO and ischemic processes of torsion on the SII index may not have been fully realized due to relatively small number of cases in this study. In addition, the fact that SII was calculated only in the ischemia phase and not in the reperfusion phase (the blood of our patients belonged to the preoperative period), which is expected to have significant parametric value changes in the IR model, may have prevented us from reaching a result with a significant difference. Therefore, prospective studies with more patients are needed. The place of SII in the prognosis of TT can be determined by comparing pre-diagnosis and postoperative complete blood counts in TT cases.

The lack of statistical significance for SII in our study may be attributed to the timing of blood sampling, which was restricted to the preoperative (ischemic) phase. Although inflammation is known to intensify during the reperfusion phase following detorsion, the primary focus of this research was to identify a reliable marker for preoperative differential diagnosis. Since the surgical decision-making process occurs before exploration, markers that change only in the post-operative period offer limited clinical utility for the initial triage of the acute scrotum.^11^,^14^ Our results suggest that in the hyperacute ischemic phase, systemic inflammatory indices may not yet diverge sufficiently to differentiate between torsion and infectious causes, reinforcing the continued reliance on scrotal DUSG and physical examination.

In the testicular torsion series of Carlos Delkodo and his team, they published that NLR is not sufficient for the diagnosis of TT and that it should be evaluated together with ultrasound.^18^ In our study, we found that scrotal USG and CRP were significant in the differential diagnosis of AS.

Monocytes have various roles in the inflammatory process, such as phagocytosis and cytokine release. It has been previously published that routine blood count and MLR ratios can be used in the differential diagnosis of TT. Unlike the studies of Bedel C et al.,^19^ we found that it is not appropriate to use the MLR ratio in the differential diagnosis of TT and EO. While C. Bedel compared children with TT and normal physical examination findings, we compared TT and EO cases. We think that the reason why our results are different is the difference between the compared groups.

In a 2019 study comparing retrospective testicular torsion with normal cases, it was stated that PLR is important in the diagnosis of testicular torsion.^20^ In 2021, Can B et al. compared TT and EO cases in their study and published results parallel to the results in our study.^21^ In our study, we did not find any significant difference in terms of PLR between the TT and EO groups. We think that PLR cannot be used in the differential diagnosis of EO in TT cases.

The present study has shown that scrotal DUSG examination is still the most valuable test in the differential diagnosis of AS. When the TT group was analyzed in itself, it was found that DUSG examination gave positive results at the rate of 89.5%; whereas, it gave positive results at the rate of 94.7% in EO cases. The value of the DUSG examination has been mentioned by previous studies.^1^,^22^-^24^ It is considered that the use of DUSG may reduce the rate of scrotal exploration; however, DUSG is operator dependent and may be difficult to perform in prepubertal patients. While the sensitivity is high in complete torsion, the false negative rate is high in incomplete torsion.^25^ CRP and DUSG are still important in the differential diagnosis after the initial evaluation of AS cases.

Our study, in line with current literature, emphasizes that DUSG remains the most reliable diagnostic tool.^25^

### Limitations:

It is a retrospective study and relatively small number of cases.

Multicenter prospective studies with a larger number of patients may be more useful in determining the use of SII in acute scrotum.

## CONCLUSION

In AS cases, scrotal DUSG examination is important in the differential diagnosis and the differential diagnosis should be supported by anamnesis, physical examination findings, and CRP. Preoperative and postoperative blood count series and SII can be used to determine the prognosis in TT cases even if it has no place in the differential diagnosis of ischemic events of the testis. More studies are needed to determine the use of SII in the differential diagnosis of AS.

### Author’s Contribution:

HM & CU: Study concept, dsesign, Literature search, Data Collection and anaylasis. Both authors are accountable for intergrity of the study and have approved the final version.
